# The Toxicity of Putrid Food

**Published:** 1921-10

**Authors:** William G. Savage

**Affiliations:** (County Medical Officer of Health, Somerset)


### THE TOXICITY OF PUTRID FOOD.

By William G. Savage, M.D. (County Medical Officer of Health, Somerset).

The quantity of food destroyed because it shows signs of decomposition is enormous. Much of it is destroyed by the activities of health officers more because it is an unsaleable article. There is no hesitation on the part of the officers of local authorities in condemning this food; there is no resistance against such action by its owners, while support for this procedure is always forthcoming without difficulty from legal tribunals and from the public generally. It is universally admitted to be unfit for food. With such unanimity, it would naturally be anticipated that there would be the clearest and strongest scientific evidence in support. It is the purpose of this article to consider how far this scientific proof of the harmfulness of tainted food is available.

If decomposing food possesses the toxicity ascribed to it, it should be easy to support the contention with evidence along at least four lines. It should be possible to cause illness in animals by feeding them with decomposing food. It should be practicable to isolate the bacteria associated with this condition, and to show that these bacteria are capable when administered by the mouth of producing prejudicial symptoms. The bio-chemist should be able to separate the products of putrefaction and prove which of them possess disease-producing properties. Lastly, it should not be difficult to point to outbreaks of food poisoning or illness in individuals caused by the ingestion of putrid material. All four lines of evidence merit consideration.

### 1. Data from the Direct Feeding of Putrid Food to Animals.

Without claiming to have made an exhaustive search through the literature, I have not come across any reports of experiments of this character. I therefore carried out an extensive series of feeding experiments, using kittens for this purpose. The results, with full details of the individual experiments, are recorded in the *Journal of Hygiene* (Vol. xx., July 1921, p. 69), to which I must refer those who wish to study the details. I have only space here to give a brief summary. In these experiments nineteen different kittens, five rabbits, and two guinea pigs were used. The feeding was mostly with very putrid food, but in some cases with food in the early stages of decomposition. In the majority the whole putrid emulsion was used, very large doses being given, but in some an endeavour was made to study the effects of the products and of the bacteria separately.

The putrid meat extracts containing both the bacteria and their products were fatal to rabbits when the method of introduction was by subcutaneous injection, but a rabbit fed with the same material remained unaffected. On the other hand, two rabbits and one kitten, injected either subcutaneously or intraperitoneally with the chemical products freed from bacteria, showed no ill effects, apart from loss of weight. The feeding experiments are mostly in agreement. In general there were no symptoms, and, apart from some loss of weight, which was rapidly regained, no physical signs. The only experiment which could lend any support to the harmfulness of putrid food was Experiment 9. In this experiment the kitten was fed on four successive days with an emulsion (containing about 60 grams of meat) from a very offensive tin of canned meat containing abundant *B. proteus*. The animal remained lively and active, without vomiting, diarrhœa, or other symptoms of illness. It, however, suddenly became ill one morning, and died seven days after the last feeding. It lost 123 grams weight over the eleven days. Post-mortem it was very thin, but there were no lesions. *B. proteus* could not be isolated. This suggested toxicity of *B. proteus* was not in accordance with the other three feeding experiments, which showed no illness, in which this organism was very abundant.

Experiment 13 may be epitomised as an example of one of the more complicated experiments. The material used was a mixture of four tins of very unsound canned fish (three salmon, one sardines). The dose fed on each day per animal was equivalent to 20 grams of the mixed solids.

Four kittens were used. Kitten *A* received the whole emulsion, Kitten *B* the sterile filtrate, Kitten *D* the emulsified bacteria without the chemical products, Kitten *C* (the control) received no fish extract. The kittens were fed on seven consecutive days with the fish extracts, all the animals receiving in addition exactly the same amount of other food; milk only being given until the end of the experimental feeding, then milk and bread. No symptoms were exhibited at any time. The weight changes (in grams) were as follows:

| | *A* | *B* | *C* | *D* |
|---|---|---|---|---|
| Weight at the onset of the feeding ... | 710 | 761 | 615 | 683 |
| Weight at end of the 7 days of feeding | 720 | 723 | 623 | 702 |
| Weight 21 days after end of the experiment ... ... ... ... ... | 1010 | 935 | 891 | 964 |
| Loss or gain of weight at end of the 7 days... ... ... ... ... | +10 | −38 | +8 | +19 |
| Gain of weight in last three weeks ... | 290 | 212 | 268 | 262 |
| Total gain in weight during the whole 28 days ... ... ... ... | 300 | 174 | 276 | 281 |
| Total gain in weight during the whole 28 days per 100 grms. of body weight | 42·4 | 22·9 | 44·9 | 41·1 |

The post-mortem appearances in Kitten *A* showed a well-nourished animal with a good deal of subcutaneous fat, fat round the kidneys, etc. Internal organs appeared perfectly normal. Only Kitten *B* showed any maintained loss of weight, but this is not a regular phenomenon, since it was not observable in Experiments 11 and 12, which were on similar lines.

In all the series of experiments the amount of putrid material consumed was enormous, each individual dose being very large, while it was given *daily* over many days. That these heroic doses of such very nauseating substances should have produced so little disturbance of nutrition or loss of weight is striking, and strongly suggests that their

**The Toxicity of Putrid Food—(*cont.*).**

toxicity is either entirely absent or of very low grade.

While, of course, it is not justifiable to assume that what is harmless to the kitten is without prejudice to man, these experiments do suggest that the toxicity can only be of low grade. They are supported by many natural-feeding experiments by man himself, for it is well known that many uncivilised races habitually consume fish, game, and other foods in a condition of definite putrefaction, while "civilised" man, in his preference for game which is "high," is following a practice only differing in degree.

2. Toxicity of Putrefactive Bacteria.

While it is scarcely correct to speak of putrefactive bacteria as if they were a definite group, undoubtedly certain organisms are found commonly associated with the putrefactive changes in foods and are capable, singly or in combination, of producing the chemical products we associate with putrefaction. The most important of such organisms are *B. proteus* (using the term to include a group of organisms) and the putrefactive anærobes such as *B. sporogenes* and *B. putrificus*. The most putrefactive of these anærobes are non-pathogenic to laboratory animals even when injected and appear to be quite harmless when fed.

The pathogenicity of *B. proteus* is higher, as many strains are decidedly pathogenic on injection under the skin, but their pathogenicity by feeding is negligible or absent. With strains isolated from cases of infantile diarrhœa, Metchnikoff and his followers set up in a few instances gastro-enteritis by prolonged feeding, while Herter and Broeck caused diarrhœa with green stools in one monkey out of three fed with a strain of this organism. There is no evidence that I can find showing that strains of this organism isolated from putrefactive material are capable of setting up illness in animals by feeding. In several of the experiments mentioned above, *B. proteus* was very abundant in the putrid foods used for feeding.

These organisms are bacteria which live in and break down dead tissue and organic matter, and are not pathogenic bacteria capable of growing and producing toxic products in a living host.

3. The Chemical Products of Putrefaction.

The study of the chemical products of putrefactive origin may be said to have passed through two phases. The first of these is the one dominated by the ptomaine hypothesis. Earlier investigators, working with a very incomplete knowledge of the decomposition products of the protein molecule, isolated a number of products of the nature of diamines from animal matter which they had allowed to putrefy for long periods and which diamines, when injected into animals, caused marked symptoms usually culminating in death. They were isolated from the late stages of putrefaction, and represented late protein degradation products. They were only produced when the food was far too nasty to run any chance of being eaten. The whole of the evidence as to their toxicity rests upon *injection* experiments, and there are no facts showing that they have any material toxicity when introduced by the mouth. Ptomaines can certainly be dismissed as having anything to do with the alleged toxicity of tainted meat.

It seems necessary to emphasise this point, since even modern textbooks dealing with the subject usually still go extensively into the properties of ptomaines and accentuate their toxicity (always by injection). The student may well be excused if he leaves the perusal with the impression that ptomaines are important disease-producing poisons with which tainted food is heavily charged, and that the consumption of such food must cause the severest symptoms, and often death.

The second phase is one widely accepted, but while more scientific in its basis is equally unconvincing. It relies for its evidence upon the fact that certain definite protein degradation products which are of considerable toxicity are manufactured by the activities of putrefactive bacteria, and that these might be produced in the early stages of putrefaction. For example, it has been shown that the poisonous bases $\beta$-amidazolethylamine and tyramine are produced by the action of putrefactive bacteria upon histidine and tyrosine, two amino-acids, early stages in the breaking down of proteins. The evidence, however, as to their poisonous properties is derived from injection, and not feeding experiments, while the fact that these poisonous bodies are produced from the cleavage of proteins by bacterial action in the *normal* intestine suggests that the human body possesses a defensive mechanism capable of dealing effectively with them.

There is no evidence that I am aware of showing that these poisonous bodies occur to putrefactive foods, and that when fed to animals they exert any poisonous action. In other words, investigations upon the chemical products of putrefactive bacilli in meat or other food in a condition in which it would be eaten (the specific food poisoning bacilli are *not* putrefactive bacilli) have failed to show any substance or group of substances capable of originating symptoms of ill-health when taken by the mouth.

It is always dangerous to make a positive deduction from negative evidence, and it may be that poisonous bodies will be isolated in the early stages of putrefactive decomposition which are toxic by the mouth and prejudicial to man. The point I want to make is that no one has so far isolated them or advanced any evidence at all as to their existence.

4. Outbreaks of Food Poisoning Due to the Consumption of Decomposing Food.

A very careful study of the literature leads to the conviction that there are no recorded outbreaks of food poisoning which have been proved to be due to the consumption of food in a putrefying condition unaccompanied by the presence of specific food poisoning bacteria or their specific toxins. Small outbreaks confined to a single case, or two or three members of one family, are usually not investigated, so it cannot be asserted that none of these could be

**The Toxicity of Putrid Food—(*cont.*).**

due to the consumption of food in an incipient state of decomposition, but certainly the connection has never been established. This aspect of the subject is dealt with in considerable detail in my book, "Food Poisoning and Food Infections, 1920," Cambridge University Press, and may be referred to for a discussion of those outbreaks which have been alleged to be due to decomposing food.

I have quite recently investigated an extensive outbreak of food poisoning, with many cases in which the food at the time of seizure was definitely decomposed, and which was probably in the early stages of decomposition when eaten. If it had not been studied with the precision of modern bacteriological methods it would inevitably have been recorded as an example of an outbreak due to putrefactive food. Investigation showed it to be due to the infection of the food with one of the specific food-poisoning organisms. It was a fairly ordinary outbreak of specific food poisoning, in which by chance (a rather rare feature) the food happened to be also tainted.

SUMMARY.

The above brief summary shows an almost entire absence of any evidence that tainted and incipiently decomposed food is harmful. The prevailing view as to its harmfulness may be perfectly justified, but it does not seem to be supported by any reliable evidence in its favour.

The firm support given to the theory of the harmfulness of tainted food is largely a matter of sentiment, supported by erroneous deductions from data which are vitiated by three prevailing fallacies, all of which have been touched upon. One is that the chemical studies upon which the theory of the harmfulness of food in a condition of putrefaction has been built up rest upon a study of the end products of putrefaction and not upon an investigation of the toxicity of such foods in stages of putrefaction when there is a possibility of their being consumed. The whole ptomaine theory, built as it is upon such foundations, is entirely unsound.

The second fallacy is that nearly the whole of the evidence as to the alleged toxicity of the products of protein decomposition is based upon experiments upon animals in which the method of introduction is by injection and not by feeding. The introduction of the products of decomposition, alien as they are to the animal economy, directly into the tissues might be expected to produce intoxication which would not occur if the product had been introduced by the mouth and been subject to the action of the digestive juices. It is well known that there are a number of substances, such as snake venoms and products of pathogenic bacteria, which are nearly harmless by the mouth but intensely toxic when introduced under the skin.

The remaining fallacy is that the alleged harmfulness of meat or other foodstuff in a condition of incipient putrefaction has usually been confused with poisoning by food infected with specifically harmful bacteria. This fallacy is kept up by the retention of the expression *ptomaine poisoning* for outbreaks of this character. There is every excuse for its retention by an uninformed lay Press, since it is so frequently used by those who should know better, but it is difficult to understand its continued use by medical men and particularly by Medical Officers of Health, in view of the abundant evidence as to its inaccuracy. Its retention and the mental attitude it connotes is very detrimental to progress. Anyone who wrote about an outbreak of typhoid fever under the name of putrid fever and gravely discussed its origin from decomposing filth would be justly stigmatised as grossly ignorant of the scientific side of his subject. To describe an outbreak of food-poisoning, which is also due to specific bacteria, as an outbreak of ptomaine poisoning, and then discuss it as if it arose from tainted or decomposed food, displays a comparable degree of ignorance.

SOME ADMINISTRATIVE AND PRACTICAL CONSIDERATIONS.

It may be advanced that a view which minimises or denies the evidence incriminating tainted meat as a cause of illness is a very damaging one administratively and will, or may, lead, if accepted, to a disregard of cleanliness and the consumption of food which is stale or possibly even tainted. It may further be contended that, whether the view of the highly dangerous condition of tainted meat is right or wrong, the conception has been of great advantage to the administrator and a powerful means whereby improvements have been effected in obtaining a cleaner, fresher, and purer food supply.

In the first place, I will freely admit that we are not in a position to say that tainted food is harmless. All we can safely say is that its evils are grossly exaggerated and that it is a minor, if not a negligible, factor in the production of toxic symptoms in man. In the second place, if food is found to be decomposed it has evidently been exposed to conditions favouring bacterial infection, and such infection may include the special bacteria associated with food infections. Confronted with decomposing food, we cannot say without detailed investigation if this is the case or not, so whatever views are held, it would be necessary and justifiable to condemn such food as an administrative action. In the third place, the stimulus needed for effective administrative action at the present time is a realisation that food-poisoning outbreaks and attacks of illness generally, from unsound food, are due to infection with specific bacteria in nearly all, if not in all, cases. The present hazy conceptions as to food-poisoning and its relationship to tainted food are merely hindrances to effective administrative action. In the end, I consider that the administrative control of our food supply will not only not be weakened, but greatly strengthened, by a recognition of the conceptions advanced here.

---

CHOLERA IN RUSSIA.—A report from Riga at the end of August states that over 78,000 cases of cholera have occurred in Russia from the beginning of this year up to August 10. Conditions in Astrakhan on the Volga are so desperate that local authorities have proposed that the whole population be transferred to Siberia and the town of Astrakhan be then set on fire.

